# Intestinal metaplasia in gallbladders: prevalence study

**DOI:** 10.1590/S1516-31802008000400004

**Published:** 2008-07-03

**Authors:** José Eduardo Vasconcelos Fernandes, Maria Isete Fares Franco, Reinaldo Kenji Suzuki, Nelson Mattos Tavares, Sansom Henrique Bromberg

**Keywords:** Lithiasis, Cholecystitis, Gallbladder, Metaplasia, Cholecystectomy, Litíase, Colecistite, Vesícula biliar, Metaplasia, Colecistectomia

## Abstract

**CONTEXT AND OBJECTIVE:**

Gallbladder cancer is usually diagnosed at a late stage and generally results in death. Discovery of predisposing factors for this neoplasia could prevent this outcome. In this study, we assess the presence of one of these factors: intestinal metaplasia in gallbladders with stones and inflammatory processes.

**DESIGN AND SETTING:**

Cross-sectional study in Hospital do Servidor Público Estadual de São Paulo.

**METHOD:**

The first 80 gallbladders from patients who underwent elective cholecystectomy between April and August 2002, presenting stones and chronic inflammation, were studied. The patients were divided into groups according to their age: CC1, from 15 to 40 years; CC2, from 41 to 60 years; and CC3, from 61 to 85 years.

**RESULTS:**

Twenty-one patients (26%) were male, while 59 (74%) were female. In the group CC1, intestinal metaplasia was present in 85.71% of the 21 patients studied; in CC2, in 79.41% of 34 patients; and in CC3, in 56.00% of 25 patients. These differences presented statistical significance (p = 0.04542).

**CONCLUSION:**

Intestinal metaplasia is extremely frequent in gallbladders with inflammation and lithiasis, especially in younger patients.

## RESUMO

**CONTEXTO E OBJETIVO:**

O câncer da vesicular biliar é diagnosticado tardiamente na maioria das vezes, levando invariavelmente a morte rápida. A detecção de fatores predisponentes ao aparecimento dessa neoplasia pode evitar esse desfecho. Avaliamos, neste estudo, a presença de um desses fatores, a metaplasia intestinal em vesículas biliares com cálculos e inflamação.

**TIPO DE ESTUDO E LOCAL:**

Estudo transversal, realizado Hospital do Servidor Público Estadual de São Paulo.

**MÉTODOS:**

Foram estudadas as primeiras 80 vesículas biliares de pacientes submetidos a colecistectomias eletivas entre abril e outubro de 2002, que apresentavam cálculos e inflamações crônicas. Os pacientes foram divididos em grupos segundo a faixa etária: CC1 de 15 a 40 anos, CC2 de 41 a 60 anos e CC3 de 61 a 85 anos.

**RESULTADOS:**

21 pacientes (26%) eram do sexo masculino, enquanto 59 (74%) do sexo feminino. No grupo CC1, foram estudados 21 pacientes e encontrou-se metaplasia intestinal em 85,17%. Já no grupo CC2 a presença de metaplasia intestinal foi de 79,41% em 34 pacientes, e no grupo CC3, de 56,00% em 25 pacientes avaliados. As diferenças tiveram significância estatística (p = 0,04542).

**CONCLUSÃO:**

A prevalência de metaplasia intestinal é extremamente alta em vesículas biliares com colecistite crônica e litíase, particularmente em pacientes jovens.

## INTRODUCTION

Lithiasis is a chronic or acute inflammatory process that is usually associated with stones (calculi). Adenocarcinoma is among the most frequent diseases of the gallbladder. Gallbladder lithiasis represents 95% of occurrences of biliary tract disease and is found worldwide. In developed countries, it affects 10% to 20% of the adult population.^1^

The consequences of stones in the gallbladder are inflammation of the organ, bile stasis and obstruction. Gallbladder stones are known to be the main risk factor for the development of gallbladder malignant neoplasia and are present in up to 75% of such cases.^2-5^

Inflammatory processes in the gallbladder have been described as the causal factor for a metaplastic response from the gallbladder epithelium. The irritation factor caused by stones produces changes in cell differentiation, thereby resulting in an adaptive response to this aggression, i.e. the formation of gastric and intestinal metaplasia. Recent studies have suggested that these epithelial changes are distinct phases of the epithelial differentiation leading to dysplasia, the lesion that ultimately precedes adenocarcinoma of the gallbladder. These studies have, as would be expected, reported increasing frequency of epithelial alterations with age.^6-8^

Some unusual findings in Brazilian anatomopathological reports and articles on metaplasia in the gallbladder epithelium, encouraged us to undertake further investigations.^9^

## OBJECTIVE

The main objective of this cross-sectional study was to assess the prevalence and clinical importance of intestinal metaplasia in gallbladders in our environment.

## METHODS

Consecutive cases of chronic inflammatory processes in gallbladders that underwent elective surgical treatment for the first time at Hospital Santa Casa de Misericórdia de Santos between April and August 2002 were studied prospectively. A total of 203 gallbladders that presented acute inflammatory processes but were free of lithiasis were excluded. Eighty consecutive gallbladders that presented lithiasis in addition to chronic inflammatory processes were included in this study. The subjects were grouped according to their age: CC1, from 15 to 40 years; CC2, from 41 to 60 years; and CC3, from 61 to 85 years.

The resected gallbladders were initially fixed in 10% formalin. Their length, maximum circumferential width and wall thickness were measured, and the numbers and characteristics of the stones found were determined. The gallbladders were then placed on Styrofoam boards, stretched, pinned down and dipped in 10% formalin. Twenty-four hours later, three 1.5 cm-long and 0.5 cm-wide blocks were cut out: one in each of the neck, body and fundus regions.^10^ These were processed to produce thin sections mounted on slides, which were stained using hematoxylin-eosin.

Intestinal metaplasia was considered to be present if goblet cells were observed in the coating epithelium or glandular epithelium in at least one of the regions studied.

For statistical analysis, a 5% level of significance (α = 0.05) was used. The chi-squared test was used to evaluate the data.

## RESULTS

The patients were between 15 and 85 years old. Overall, twenty-one patients (26%) were male, while 59 (74%) were female. The male/female ratio was 1:2.8. The female predominance was 71.43% in the CC1 group, 67.65% in CC2 and 84% in CC3, i.e. according to age. One patient was Asian, one was black and the other 78 were white.

Among the 80 gallbladders analyzed, the mean length was 79.35 mm, the maximum circumferential width was 46.68 mm and the wall thickness was 4.11 mm. The stones were predominantly of mixed type (57.5%). The cholesterol and pigmented types appeared in smaller numbers (23.5% and 18.75% respectively). The cholesterol type was more frequently associated with intestinal metaplasia (84.21%), although no statistically significant difference was found.

With regard to topographic distribution, the intestinal metaplasia was diffusely present in all regions of the gallbladder, although more predominant in the fundus and neck when found focally, in all groups, independently of age.

**Figure 1:**
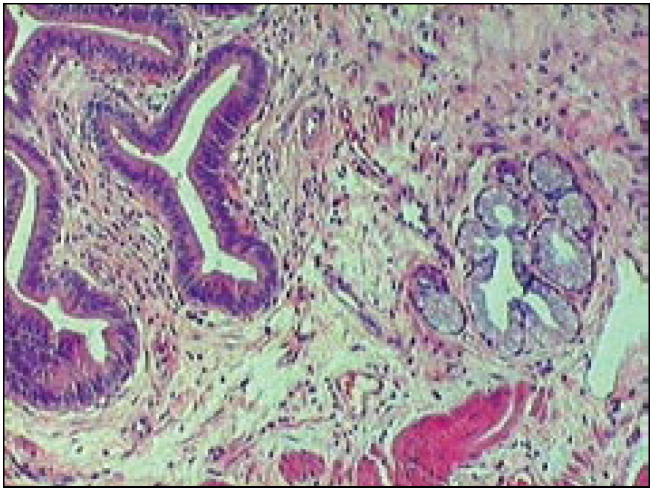
Focal area of intestinal metaplasia in gallbladder with lithiasis (hematoxylin-eosin, 200 x).

**Figure 2:**
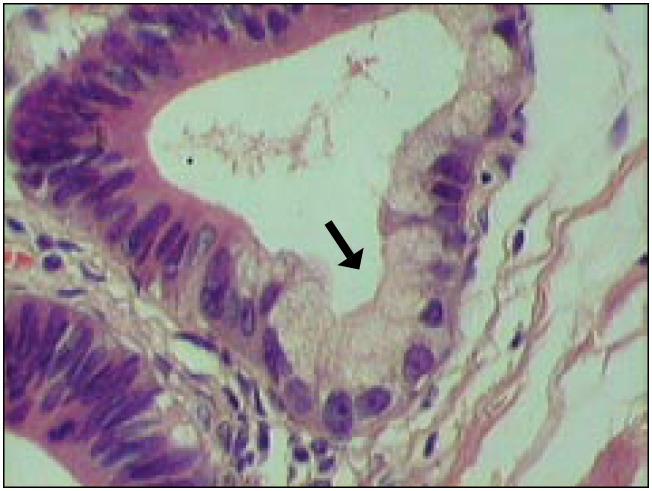
Adenocarcinoma of gallbladder: atypical glandule coated with cylindrical epithelial cells and goblet cells with pleomorphic hyperchromatic cells (detail) (hematoxylin-eosin 400 x).

Among the patients in the CC1 group, 18 (85.71%) out of 21 presented intestinal metaplasia in at least one region of the gallbladder that was examined. In CC2 and CC3, the frequency of this epithelial transformation was 79.41% (out of 34 patients) and 56.00% (out of 25 patients), respectively. The differences in frequency of intestinal metaplasia were statistically significant (p = 0.04542) among the three groups (Table 1, Figure 1 and Figure 2).

**Table 1:** Frequency of intestinal metaplasia in gallbladders, distributed among the groups CC1, CC2 and CC3

Intestinal metaplasia	Groups			
	CCI	CC2	CC3	Total
Absent	03	07	11	21
	14.29%	20.59%	44.00%	26.20%
Present	18	27	14	59
	85.71%	79.41%	56.00%	73.80%
**Total**	**21**	**34**	**25**	**80**

*χ^2^ = 6.18; p = 0.04542.*

## DISCUSSION

The high frequency of intestinal metaplasia in gallbladders with stones (73.80%) found in our study contrasts with what was observed by Jukemura (5.4%).^9^ Our findings were also higher than those of studies conducted in other countries such as India (15.5% of 140 gallbladders),^11^ the United States (9.8% of 400 gallbladders),^8^ Canada (10.8% of 277 gallbladders)^6^ and Japan (30.6% of 1,000 gallbladders and 425 of 2,024 gallbladders).^7,12^

Gastric and intestinal metaplasia in the gallbladder are considered by many authors to be the initial phase of the inflammatory response to the aggression caused by the stones. This could trigger a sequence of events that would result in malignant transformation of the gallbladder.^13-15^ According to these authors, the presence of intestinal metaplasia is progressive and it increases in frequency with age. This can be explained by the increasing length of contact of the stones with the mucosa. However, recent findings from studies on early gallbladder neoplasia^16,17^ have demonstrated that, in addition to the genetic factor, there could be some unknown carcinogenic substance in the bile that could also be involved in the genesis of gallbladder cancer. This unknown carcinogenic factor could originate from food. These studies have also shown histological alterations preceding cholelithiasis.

Our study demonstrated significantly greater intestinal metaplasia in the CC1 group than in CC2 and CC3. The explanation for this higher frequency of metaplasia and the earlier appearance of this histological alteration seems to be the change in diet that has been taking place in our country, particularly over the last decade, since fast food full of carbohydrates and fats has become popular among young people. This has caused an increase in the number of obese children and adolescents, as was demonstrated in a recent study conducted in Santos, Brazil. In that study, it was found that out of 10,821 elementary school students, 33.71% were obese.^18^

The most important studies on alterations of the gallbladder due to metaplasia are now two decades old. They showed that such alterations were increasingly common with advancing age. However, a study one decade ago was unable to demonstrate this directly proportional relationship between age and the presence of intestinal metaplasia.^9^ Our study demonstrates a higher frequency of intestinal metaplasia among young patients, which strengthens the hypothesis that the external factors have changed over the generations, with evolution of the histological alterations, notably intestinal metaplasia of the gallbladder.

Gallbladder cancer is the most common malignant tumor of the biliary tract and the fifth most frequent in the digestive tract.^19^ Eighty to ninety percent of the patients with this neoplasia will present associated gallbladder stones.^20^ Since Järvis and Laurén's description showing that the presence of intestinal metaplasia in the nasal mucosa was a predisposing factor for nasal cavity cancer, an association between intestinal metaplasia and cancer has been shown in many other organs.^21^

Studies have shown that intestinal metaplasia is a predisposing factor for the development of gallbladder cancer.^22-24^ The high frequency of an association between intestinal metaplasia and gallbladder stones and the chronic inflammatory process in the gallbladder found in our study, together with the low morbidity and mortality of present-day video cholecystectomy procedures, suggest that patients with gallbladder stones should undergo elective surgery even when no symptoms are presented. This has also been stated by other authors.^8^

Thus, we share the view of other researchers who are in favor of early surgical procedures.^9,25^ For patients who are unwilling to undergo surgical procedures, we suggest that follow-ups should be carried out more frequently, in an attempt to diagnose this neoplasia earlier. This noninvasive management ought to be studied so that the natural history of gallbladder cancer can be better understood.

## CONCLUSION

From our study, we can conclude that intestinal metaplasia presents extremely high frequency among patients with chronic cholecystitis with stones, particularly among young patients.
